# Clinical outcomes of hospitalized COVID-19 patients with renal injury: a multi-hospital observational study from Wuhan

**DOI:** 10.1038/s41598-021-94570-1

**Published:** 2021-07-26

**Authors:** Kehong Chen, Yu Lei, Yani He, Fei Xiao, Yan Yu, Xiaodong Lai, Yang Liu, Jiang Wang, Huanzi Dai

**Affiliations:** 1 Department of Nephrology, Daping Hospital, Army Medical University, Changjiang Branch Road 10, Yu Zhong district, Chongqing, 400042 China; 2 Department of Critical Care Medicine, Taikang Tongji Hospital, Wuhan, China; 3 Department of Infectious Disease, Huo Shen Shan Hospital, Wuhan, China; 4 Department of Anaesthesiology, Daping Hospital, Army Medical University, Chongqing, China; 5 Department of Critical Care Medicine, The People’s Hospital of Wuhan University, Wuhan, China; 6 Department of Critical Care Medicine, The Thirteenth People’s Hospital of Chongqing, Chongqing, China; 7 Department of Infectious Disease, Taikang Tongji Hospital, Wuhan, China; 8 Department of Neurology, The No. 988 Hospital of Joint Logistic Support Force, Zhengzhou, China; 9 Department of Cardiology, Xinqiao Hospital, Army Medical University, Chongqing, China

**Keywords:** Nephrology, Risk factors, Medical research, Outcomes research

## Abstract

Renal injury is common in patients with coronavirus disease 2019 (COVID‐19). We aimed to determine the relationship of estimated glomerular filtration rate (eGFR) and acute kidney injury (AKI) with the characteristics, progression, and prognosis of COVID-19 in-patients. We retrospectively reviewed 1851 COVID-19 patients admitted to 3 hospitals in Wuhan, China. Clinical, laboratory, radiological, treatment, complication, and outcome data were analyzed. Patients were stratified according to levels of eGFR (≥ 90 vs. 60–89 vs. < 60 mL/min/1.73 m^2^). The risk of reaching the composite endpoint—intensive care unit admission, invasive ventilation, or death—was compared. On admission, 25.5% patients had renal impairment (eGFR < 90 mL/min/1.73 m^2^), but only 2.6% patients had chronic kidney disease (CKD). The overall in-hospital AKI incidence was 6.7%. Severe illness and comorbidities (hypertension, diabetes, CKD, and cardiovascular/cerebrovascular diseases) were more common among patients with low eGFR (< 90 mL/min/1.73 m^2^). Despite the more frequent use of intensive oxygen therapy, continuous blood purification, and glucocorticoid treatment, the prognosis of these patients was unsatisfactory, with the incidence of the composite endpoint (15.4% vs. 19.6% vs. 54.5%; P = 0.000) and complications (AKI, respiratory failure, cardiac injury, coagulation disorders, sepsis, etc.) increasing with decreasing eGFR. Kaplan–Meier survival analysis revealed that patients with eGFR < 90 mL/min/1.73 m^2^ or AKI had significantly escalated risks of reaching the composite endpoint. Multivariate regression analysis showed that renal insufficiency (eGFR < 60 mL/min/1.73 m^2^) on admission and in-hospital AKI independently predicted poor prognosis among COVID-19 in-patients. And renal impairment on admission was a greater predictor of poor prognosis in non-elderly patients than that in elderly patients. Early and continuous renal-function monitoring and early AKI diagnosis are necessary to predict and prevent the progression of COVID-19.

## Introduction

Coronavirus disease 2019 (COVID‐19) is a pandemic viral disease caused by severe acute respiratory syndrome coronavirus 2 (SARS-CoV-2), which broke out in Wuhan, China, in December 2019 and spread worldwide^1^. As of May 10, 2021, there have been 158,334,639 confirmed COVID-19 cases and 3,293,120 deaths worldwide (announced by World Health Organization [WHO]). Infection with SARS-CoV-2 not only leads to severe acute respiratory syndrome but also causes damage to the kidneys^2^, heart^3^, and other organs. 13.1%–14.4% of COVID-19 patients had some renal injury markers on admission, including proteinuria, hematuria, and elevated serum creatinine (SCr) and blood urea nitrogen (BUN). Patients with markers of kidney impairment were at a higher risk for in-hospital death^4^. Some studies have found that CKD is associated with the risk of severe COVID-19 symptoms^5,6^, and subsquent death^7^ in patients with COVID-19. And there was a significant increase in mortality in CKD stages 3–5^8^.

Acute kidney injury (AKI) is an important complication of COVID-19, occurring in 0.5%–9% of all cases and 10%–30% of intensive care unit (ICU) or critically ill patients^9–13^. Although it has been reported that SARS-CoV-2 infection might not result in AKI or aggravate chronic kidney disease (CKD)^14^, a large amount of evidence demonstrates that AKI at an early stage is a negative prognostic indicator for COVID-19^15,16^. Moreover, severe acute kidney injury associated with progression of chronic kidney disease after critical COVID-19^17^. Although a poorer outcome of COVID-19 in patients with CKD, reduced renal function at admission and when complicated by AKI have already been reported by several groups in different settings^18^, our study collected a large number of original data from Wuhan, the first COVID-19 outbreak point, which may be beneficial to provide scientific information for the further study of epidemic outbreak model.

In this study, we retrospectively analyzed an outbreak of COVID-19 in 3 hospitals in Wuhan, China, which is the first COVID-19 outbreak place. The epidemiological, clinical, laboratory, and radiological characteristics as well as the treatment, complication, and outcome data of 1851 COVID-19 patients were stratified according to the eGFR on admission and reviewed. We aimed to confirm the association between renal injury and in-hospital prognosis among patients with COVID-19 in the first COVID-19 outbreak site.

## Results

### Patient characteristics

A total of 1851 patients hospitalized with confirmed COVID-19 from 3 hospitals (1412 patients from Taikang Tongji Hospital, 335 patients from Huo Shen Shan Hospital, and 104 patients from Renmin Hospital of Wuhan University) were included in this study. To elucidate the clinical implications of renal dysfunction in COVID-19 patients, we stratified the patients into three groups according to their baseline eGFR as follows: 1379 patients (74.5%), ≥ 90 mL/min/1.73 m^2^ (high eGFR group, HEG, normal renal function); 360 patients (19.4%, ), 60–89 mL/min/1.73 m^2^ (medium eGFR group, MEG, mild renal impairment); and 112 patients (6.1%), ˂60 mL/min/1.73 m^2^ (low eGFR group, LEG, moderate/severe renal function impairment). The characteristics of the 1851 COVID-19 patients are presented in Table 1. The median age was 62 years (IQR, 51–70 years), and 889 (48.0%) patients were male. Severe cases accounted for 37.9% of the study population. The most common symptoms on admission were fever (1099 patients, 59.3%) and cough (1044 patients, 56.4%). Asymptomatic patients accounted for 2.1% of the study population (Supplementary Table 1). The median duration from symptom onset to admission was 25 days (IQR, 14–38 days). Comorbidities were present in half the patients (50.5%), with hypertension (697 patients, 37.7%) and diabetes (310 patients, 16.8%) being the most prevalent comorbidities. Only 2.6% patients had a history of CKD.

**Table 1:** Clinical characteristics of COVID-19 patients stratified by eGFR levels.

Variable	All patients (N = 1851)	eGFR, ml/min/1.73 m^2^			P value^a^
		≥ 90 (N = 1379)	60–89 (N = 360)	< 60 (N = 112)	
N (%)	1851	1379 (74.5)	360 (19.4)	112 (6.1)	
Age (years)	62 (51,70)	59 (48, 67)	73 (63,81)	72 (64,81)^#,&^	0.001
Male sex, n/N (%)	889/1851 (48.0)	650/1379 (47.1)	180/360 (50.0)	59/112 (52.6)	0.373
BMI (kg/m^2^)	23.42 (21.25,25.51)	23.32 (21.22,25.39)	23.56 (21.22,26.03)	23.71 (22.03,25.83)	0.579
Systolic pressure on admission (mm Hg)	130 (120, 142)	130 (120, 141)	132 (120, 145)^#^	131.5 (120, 145)^&^	0.000
Diastolic pressure on admission (mm Hg)	80 (74, 88)	80 (73.5, 88)	80 (74, 88)	80 (75, 90)	0.183
Mean arterial pressure on admission (mm Hg)	96.67 (89.33,106)	96.66 (90,104.66)	98.5 (91.66,107.33)^#^	95.33 (88.08,105.33)^&^	0.008
**Disease severity, n/N (%)**					
Non-severe	1149/1851 (62.07)	930/1379 (67.44)	188/360 (52.22)^#^	31/112 (27.67)^#,&^	0.000
Severe	700/1851 (37.87)	447/1379 (32.41)	172/360 (47.78) ^#^	81/112 (72.32) ^#,&^	0.000
Time from symptom onset to admission (days)	25 (14, 38)	26 (14, 39)	21 (14, 36)	17 (8, 36)^#^	0.017
**Comorbidities, n/N (%)**					
Any	934/1851 (50.45)	598/1379 (43.36)	238/360 (66.11) ^#^	98/112 (87.5) ^#,&^	0.000
Hypertension	697/1851 (37.67)	424/1379 (30.75)	191/360 (53.05)^#^	82/112 (73.21) ^#,&^	0.000
Diabetes	310/1851 (16.77)	200/1379 (14.50)	73/360 (20.27)^#^	37/112 (33.03)^#,&^	0.000
Coronary heart disease	199/1851 (10.75)	104/1379 (7.54)	62/360 (17.22)^#^	33/112 (29.46)^#,&^	0.000
Chronic obstructive pulmonary disease	78/1847 (4.22)	53/1379 (3.84)	18/360 (5.00)	7/112 (6.25)	0.342
Cerebrovascular disease	129/1849 (6.97)	71/1379 (5.15)	41/360 (11.38)^#^	17/112 (15.17)^#^	0.000
Chronic kidney disease	48/1849 (2.59)	6/1379 (0.44)	8/360 (2.22) ^#^	34/112 (30.35) ^#,&^	0.000
Hepatitis B infection	27/1848 (1.46)	18/1379 (1.31)	6/360 (1.67)	3/112 (2.67)	0.512
Cancer	38/1849 (2.05)	29/1379 (2.10)	5/360 (1.38)	4/112 (3.57)	0.347

Data are expressed as median (interquartile range) or no./total no. (%). ^a^P values were calculated using the Kruskal–Wallis test or the chi-square test.

COVID-19, coronavirus disease 2019; eGFR, estimated glomerular filtration rate; BMI, body mass index.

^#^ vs GFR ≥ 90 ^&^ vs 60 ≤ GFR < 90.

Compared with patients in the HEG (≥ 90 mL/min/1.73 m^2^), patients in the MEG and LEG were older, had a higher prevalence of severe illness, were more likely to experience shortness of breath and chest distress/palpitations, and were less likely to have fever and sputum production. Moreover, comorbidities, including hypertension, diabetes, coronary heart disease, cerebrovascular disease, and CKD, were present more often among patients in the MEG and LEG.

### Laboratory and radiological findings

Table 2 shows the laboratory and radiological findings on admission. In the overall study population, the median values of all laboratory indicators were within the normal range. Of the 1463 CT scans that were performed on admission, 87.6% revealed abnormal results.

**Table 2:** Laboratory and radiological findings of COVID-19 patients stratified according to eGFR levels.

Variable	All patients (N = 1851)	eGFR, ml/min/1.73 m^2^			P value^a^
		≥ 90 (N = 1379)	60–89 (N = 360)	< 60 (N = 112)	
**Laboratory findings at admission**					
**Hematological**					
Leukocyte Count, × 10^9^/L	5.84 (4.74,7.13)	5.75 (4.7, 7.0)	6 (4.8,7.22)	6.715 (5.52,9.32)	0.060
˂ 3.5**– n/N (%)**	95/1841 (5.16)	68/1370 (4.96)	23/359 (6.40)	4/112 (3.57)	0.412
> 9.5**– n/N (%)**	169 /1841 (11.26)	103/1370 (7.51)	40/359 (11.14)^#^	26/112 (23.21)^#,&^	0.000
Lymphocyte Count, × 10^9^/L	1.53 (1.12,2)	1.6 (1.2, 2.07)	1.33 (0.99,1.77)^#^	1.12 (0.62,1.79)^#^	0.000
˂ 1.1**– n/N (%)**	433/1841 (23.51)	264/1370 (19.27)	114/359 (31.75)^#^	55/112 (49.10)^#,&^	0.000
Platelet Count, × 10^9^/L	216 (176, 265)	220 (183, 270)	209 (165,257)^#^	196 (144,237)^#,&^	0.000
˂ 125**– n/N (%)**	147/1839 (7.99)	88/1368 (6.43)	32/359 (8.91)	27/112 (24.10)^#,&^	0.000
> 350**– n/N (%)**	118/1839 (6.41)	89/1368 (6.50)	24/359 (6.68)	5/112 (4.46)	0.685
Hemoglobin, g/L	120 (109,132)	121 (111, 133)	117 (105,129)^#^	109 (89,124) ^#,&^	0.000
**Biochemical**					
Creatinine, μmol/L	56.34 (46.28,69.53)	51.4 (43.21, 60.31)	73.61 (65.3,84.7)^#^	126.88 (97.42,181.7)^#,&^	0.000
> 104**– n/N (%)**	87/1851 (4.70)	0/1379 (0)	9/360 (2.5)	78/112 (69.64)^&^	0.000
Peak creatinine, μmol/L	59.99 (49.54,74.83)	55.23 (47, 64.87)	77.75 (67.14,89.68) ^#^	145.6 (106.59,212.03)^#,&^	0.000
Urea nitrogen, mmol/L	4.9 (4.03,6.1)	4.61 (3.84, 5.57)	5.71 (4.6,7.17)^#^	11.03 (7.76,15.40)^#,&^	0.000
> 7.2**– n/N (%)**	249/1851 (13.45)	73/1379 (5.29)	86/360 (23.88)^#^	90/112 (80.35)^#,&^	0.000
Albumin, g/L	37.73 (34.26,40.65)	38.3 (35.1,41.03)	36.22 (32.16,39.47) ^#^	33.88 (30.05,37.66)^#,&^	0.000
˂ 35**– n/N (%)**	547/1845 (29.64)	334/1374 (24.30)	150/360 (41.66)^#^	63/111 (56.75)^#,&^	0.000
Total bilirubin, mmol/L	10.62 (8.32,13.71)	10.58 (8.32, 13.37)	10.78 (8.32,14.94)	10.58 (8.28,15.87)	0.256
> 20.5**– n/N (%)**	113/1844 (6.12)	65/1374 (4.73)	32/360 (8.88)^#^	16/110 (14.54)^#^	0.000
Alanine aminotransferase, U/L	21.81 (14.58,36.7)	18.83 (11.65,30.6)	21.11 (14.27,32.96) ^#^	22.64 (14.86,38.05) ^#^	0.000
> 45**– n/N (%)**	333/1844 (18.05)	256/1374 (18.63)	59/360 (16.38)	18/110 (16.36)	0.549
Aspartate aminotransferase, U/L	20.03 (15.08,28.81)	19.42 (14.49, 27.65)	21.41 (16.97,31.28) ^#^	24.4 (17.65,40.79)^#^	0.000
> 40**– n/N (%)**	181/1425 (12.70)	112/1046 (10.70)	44/284 (15.49)	25/95 (26.31)^#^	0.000
Lactate dehydrogenase, U/L	178.45 (149.28,221.01)	173.58 (145.11,212.06)	194.1 (161.93,234.81) ^#^	218.5 (164.81,367.06)^#^	0.008
> 271**– n/N (%)**	251/1851 (13.56)	147/1379 (10.65)	63/360 (17.5)^#^	41/112 (36.60)^#,&^	0.000
Creatine kinase-MB, U/L	8.15 (6.2,10.87)	8.04 (6.3, 10.76)	8.37 (6.11,10.78)	8.48 (5.87,11.2)	0.528
> 24**– n/N (%)**	32/1232 (2.59)	21/945 (2.22)	4/226 (1.76)	7/61 (11.47)^#,&^	0.001
Myoglobin, ng/mL	21 (13.2,33.33)	21 (11.4, 26.56)	29.16 (21,47.87)^#^	53.32 (29.31,253.44)^#^	0.000
> 65.8**– n/N (%)**	113/1001 (11.28)	47/741 (6.34)	37/199 (18.59)^#^	29/61 (47.54)^#,&^	0.000
High-sensitivity cardiac Troponin I, ng/mL	0.01 (0.01, 0.034)	0.01 (0, 0.02)	0.03 (0.01, 0.069) ^#^	0.058 (0.016, 0.149) ^#,&^	0.000
> 0.04**– n/N (%)**	215/1015 (21.18)	104/754 (13.79)	77/203 (37.93)^#^	34/58 (58.62)^#,&^	0.000
**Infection-related indices**					
C-reactive protein, mg/L	0.79 (0.5,8.18)	0.5 (0.5, 5)	1.69 (0.5,11.28)	10.9 (0.74,72.75)^#,&^	0.000
> 10**– n/N (%)**	399/1743 (22.89)	252/1288 (19.56)	92/346 (26.58)^#^	55/109 (50.45)^#,&^	0.000
Interleukin-6, pg/mL	2.99 (1.5,8.01)	2.4 (1.5, 5.415)	6.18 (2.38,13.89)^#^	10.94 (4.94,36.63)^#^	0.000
> 7**– n/N (%)**	403/1499 (26.88)	226/1128 (20.03)	127/289 (43.94)^#^	50/82 (60.97)^#,&^	0.000
Procalcitonin, ng/mL	0.05 (0.03,0.08)	0.04 (0.03, 0.07)	0.06 (0.04,0.12)^#^	0.16 (0.07,0.57)^#^	0.030
≥ 0.05**– n/N (%)**	618/1368 (45.17)	376/1011 (37.19)	164/261 (62.83)^#^	78/96 (81.25)^#,&^	0.000
≥ 0.5**– n/N (%)**	71/1368 (5.19)	29/1011 (2.86)	16/261 (6.13)	26/96 (27.08) ^#,&^	0.000
**Coagulation function**					
Prothrombin time, s	12.22 (11.4,13.1)	12.2 (11.5, 13)	12.6 (11.7,13.5)	13.07 (12,14.825)^#,&^	0.000
˂ 9.4**– n/N (%)**	0	0	0	0	\
> 12.5**– n/N (%)**	560/1339 (41.82)	363/973 (37.30)	141/276 (51.08)^#^	56/90 (62.22)^#^	0.000
Activated partial thromboplastin time, s	30.2 (27.6,33)	30.6 (28.2, 33.2)	30.36 (28.08,33.03)	31.34 (28.83,34.58)	0.175
˂ 25.1**– n/N (%)**	89/1338 (6.65)	65/972 (6.68)	16/276 (5.79)	8/90 (8.88)	0.589
> 36.5**– n/N (%)**	137/1338 (10.23)	95/972 (9.77)	25/276 (9.05)	17/90 (18.88)^#,&^	0.018
Fibrinogen, mg/dL	276 (217,336)	276 (227, 334)	302 (245,346)^#^	306 (251,390)^#^	0.000
˂ 238**– n/N (%)**	372/1336 (27.84)	296/971 (30.48)	54/276 (19.56)^#^	22/89 (24.71)	0.000
> 498**– n/N (%)**	40/1336 (2.99)	29/971 (2.98)	8/276 (2.89)	3/89 (3.37)	0.922
D-dimer, ng/mL	63 (2,304)	75.5 (28, 340.5)	155.5 (60,451.5)^#^	336 (116.5,655.25)^#,&^	0.000
≥ 500**– n/N (%)**	185/932 (19.84)	113/659 (17.14)	45/201 (22.38)	27/72 (37.5)^#,&^	0.000
**Urinalysis**					
**Proteinuria– n/N (%)**					
Negative	989/1223 (80.86)	786/916 (85.80)	175/240 (72.91)	28/67 (41.79)^#,&^	0.000
Positive	234/1223 (19.13)	130/916 (14.19)	65/240 (27.08)^#^	39/67 (58.21)^#,&^	0.000
+	211/1223 (17.25)	124/916 (13.53)	59/240 (24.58)^#^	28/67 (41.79)^#,&^	0.000
+ + ~ + + +	23/1223 (1.88)	6/916 (0.65)	6/240 (2.5)^#^	11/67 (16.41)^#,&^	0.000
**Hematuria– n/N (%)**					
Negative	847/1219 (69.48)	652/913 (71.41)	162/239 (67.78)	33/67 (49.25)^#,&^	0.001
Positive	372/1219 (30.52)	261/913 (28.59)	77/239 (32.22)	34/67 (50.75)^#,&^	0.001
+	307/1219 (25.18)	223/913 (24.42)	63/239 (26.35)	21/67 (31.34)	0.409
+ + ~ + + +	65/1219 (5.33)	38/913 (4.16)	14/239 (5.85)	13/67 (19.40)^#,&^	0.000
**Chest computed tomography findings-– n/N (%)**					
Abnormalities on chest CT	1282/1463 (87.63)	942/1079 (87.30)	273/304 (89.80)	67/80 (83.75)^&^	0.030
Sub-pleura	117/1463 (8.00)	102/1079 (6.97)	12/304 (3.95)^#^	3/80 (3.75)^#^	0.003
Unilateral	129/1463 (8.82)	107/1079 (7.31)	19/304 (6.25)	3/80 (3.75)	0.036
Bilateral	1036/1463 (70.81)	733/1079 (67.93)	242/304 (79.61)^#^	61/80 (76.25)^#^	0.000

Data are expressed as median (interquartile range) or no./total no. (%). ^a^P values were calculated using the Kruskal–Wallis test or chi-square test.

COVID-19, coronavirus disease 2019; eGFR, estimated glomerular filtration rate.

^#^ vs GFR ≥ 90 ^&^ vs 60 ≤ GFR < 90.

Compared with patients in the HEG, patients in the MEG and LEG showed significantly elevated cardiac injury indicators (high-sensitivity cardiac troponin I, myoglobin, and lactate dehydrogenase), liver injury indicators (alanine and aspartate aminotransferases), inflammation-related indicators (C-reactive protein, interleukin-6, and procalcitonin), and coagulation function indicators (prothrombin time, fibrinogen, and d-dimer), and significantly decreased platelet count, hemoglobin, and albumin. Unlike patients in the HEG, patients in the MEG and LEG tended to have bilateral pneumonia rather than subpleural or unilateral pneumonia.

### Treatment, complications, and clinical outcomes

The treatment, complications, and in-hospital clinical outcomes of the patients are summarized in Table 3. Of the 1851 patients, 58.9% patients required oxygen support in the hospital. The most intense level was recorded, including low-flow (46.7%) and high-flow nasal cannula (HFNC, 6.2%) oxygen inhalation, noninvasive mechanical ventilation (NMV, 1.9%), and invasive mechanical ventilation (IMV, 4.1%). Only 1.1% patients required continuous renal replacement therapy (CRRT). Extracorporeal membrane oxygenation was used in 2 patients. Antiviral therapy was the most common treatment (64.7%), followed by antibiotic therapy (41.4%) and glucocorticoids (11.9%). Renin-angiotensin system (RAS) inhibitors and diuretics were administered to 10.9% and 10.2% patients, respectively. In addition, 23 (1.3%), 44 (2.5%), and 16 (0.9%) patients were treated with tocilizumab, umbilical cord mesenchymal stem cells, and convalescent plasma, respectively. Coagulation disorder (66.2%) was the most frequent complication, followed by anemia, hypoproteinemia, electrolyte disturbances, acute cardiac injury, respiratory failure, acidosis, and ARDS. A total of 1690 patients were followed up for a median of 16 days, during which 90.7% (1532) patients were discharged, 4.9% (83) patients died, and 4.4% (75) patients remained hospitalized. The remaining patients (161) were lost to follow-up.

**Table 3:** Treatment, complications, and clinical outcomes of COVID-19 patients stratified according to eGFR levels.

Variable	All patients (N = 1851)	eGFR, ml/min/1.73 m^2^			P value^a^
		≥ 90 (N = 1379)	60–89 (N = 360)	< 60 (N = 112)	
**Treatment in hospital, n/N (%)**					
Oxygen therapy	1091/1851 (58.94)	791/1379 (57.36)	222/360 (61.67)	78/112 (69.64) ^#^	0.020
Nasal duct/mask	865/1851 (46.73)	659/1379 (47.79)	170/360 (47.22)	36/112 (32.14)^#,&^	0.006
High-flow nasal cannula	114/1851 (6.16)	76/1379 (5.51)	24/360 (6.67)	14/112 (12.50)^#,&^	0.011
Noninvasive mechanical ventilation	36/1851 (1.94)	20/1379 (1.45)	7/360 (1.94)	9/112 (8.04)^#,&^	0.000
Invasive mechanical ventilation	76/ 1851 (4.11)	36/1379 (2.61)	21/360 (5.83)	19/112 (16.96)^#,&^	0.000
Continuous renal replacement therapy	21/1733 (1.21)	7/1283 (0.54)	4/345 (1.15)	10/105 (9.52)^#,&^	0.000
Extracorporeal membrane oxygenation	2/1851 (0.11)	2/1379 (0.15)	0/0	0/0	-
Antibiotic therapy	713/1733 (41.14)	500/1284 (38.94)	149/344 (43.31)	64/105 (60.95)^#,&^	0.000
Antiviral therapy	1121/1733 (64.68)	821/1283 (63.99)	225/345 (65.21)	75/105 (71.42)	0.301
Glucocorticoids	204/1721 (11.85)	122/1274 (9.57)	45/343 (13.11)	37/104 (35.57)^#,&^	0.000
RAS inhibitors^b^	188/1729 (10.87)	111/1281 (8.66)	54/343 (15.74)^#^	23/105 (21.90)^#^	0.000
Diuretics	177/1731 (10.22)	91/1282 (7.09)	53/344 (15.40)^#^	33/105 (31.42)^#,&^	0.000
Tocilizumab	23/1728 (1.33)	14/1279 (1.09)	6/344 (1.74)	3/105 (2.85)	0.169
Umbilical cord mesenchymal stem cells	44/1728 (2.54)	32/1282 (2.49)	10/342 (2.92)	2/104 (1.92)	0.854
Convalescent plasma	16/1731 (0.92)	7/1285 (0.54)	8/341 (2.34)	1/105 (0.95)	0.11
**Complications, n/N (%)**					
Any complication	1372/1747 (78.53)	976/1293 (75.48)	296/348 (85.06) ^#^	100/106 (94.34) ^#,&^	0.000
Respiratory failure	139/1742 (7.98)	71/1290 (5.50)	35/346 (10.11)^#^	33/106 (31.13)^#,&^	0.000
Acute respiratory distress syndrome	80/1742 (4.59)	39/1290 (3.02)	20/346 (5.78)	21/106 (19.81)^#,&^	0.000
Acute heart failure	42/1742 (2.41)	14/1290 (1.08)	13/346 (3.75)^#^	15/106 (14.15)^#,&^	0.000
Acute cardiac injury	300/1663 (18.03)	151/1239 (12.18)	98/326 (30.06)^#^	51/98 (52.04)^#,&^	0.000
Acute kidney injury	115/1708 (6.67)	74/1266 (5.85)	23/340 (6.76)	18/102 (17.65) ^#,&^	0.000
Stage 1	70/1708 (4.10)	43/1266 (3.40)	13/340 (3.82)	14/102 (13.73) ^#,&^	0.000
Stage 2	26/1708 (1.52)	20/1266 (1.58)	4/340 (1.18)	2/102 (1.96)	0.806
Stage 3	18/1708 (1.05)	10/1266 (0.79)	6/340 (1.76) ^#^	2/102 (1.96) ^#^	0.039
Coagulation disorders	906/1368 (66.22)	619/999 (61.96)	213/280 (76.07)^#^	74/89 (83.14)^#,&^	0.000
Sepsis	38/1742 (2.18)	20/1290 (1.55)	13/346 (3.76)	5/106 (4.71)^#,&^	0.006
Shock	44/1742 (2.52)	17/1290 (1.31)	13/346 (3.75)	14/106 (13.20)^#,&^	0.000
Septic shock	29/1742 (1.66)	20/1290 (1.55)	9/346 (2.60)	0/106 (0.00)	1.000
Secondary infection	18/1742 (1.03)	12/1290 (0.93)	3/346 (0.86)	3/106 (2.83)	0.168
Anemia	600/1841 (32.59)	391/1370 (28.54)	147/359 (40.95)^#^	62/112 (55.36)^#,&^	0.000
Hypoproteinemia	507/1743 (29.08)	304/1289 (23.58)	145/348 (41.66)^#^	58/106 (54.71)^#,&^	0.000
Electrolyte disturbances	318/1695 (18.76)	199/1250 (15.92)	75/340 (22.05)	44/105 (41.90)^#,&^	0.000
Acidosis	81/1713 (4.72)	35/1268 (2.76)	22/341 (6.45)^#^	24/104 (23.07)^#,&^	0.000
**Clinical outcome at data cutoff, n/N (%)**					
Discharge from hospital	1532/1690 (90.65)	1174/1253 (93.70)	299/336 (88.98)	59/101 (58.41) ^#,&^	0.000
Hospitalization	75/1690 (4.44)	45/1253 (3.59)	14/336 (4.16)	16/101 (15.84) ^#,&^	0.000
Death	83/1690 (4.91)	30/1253 (2.38)	25/336 (7.44)^#^	28/101 (27.72)^#,&^	0.000
Intensive care unit admission	309/1690 (18.28)	191/1253 (15.24)	65/336 (19.34)	53/101 (52.48)^#,&^	0.000
Invasive mechanical ventilation	76/ 1690 (4.50)	36/1253 (2.87)	21/336 (6.25)^#^	19/101 (18.81)^#,&^	0.000
Composite endpoint^c^	314/1690 (18.58)	193/1253 (15.40)	66/336 (19.64)	55/101 (54.46)^#,&^	0.000
Time from symptom onset to composite endpoint (days)	21 (13,33.5)	24 (15,38.5)	19 (13,27)^#^	16 (11,30)^#^	0.015

Data are expressed as no./total no. (%).

^a^P values were calculated using the chi-square test.

^b^RAS inhibitors included angiotensin-converting-enzyme inhibitors and angiotensin receptor blockers. RAS, renin-angiotensin system.

^c^The composite endpoint comprised admission to the intensive care unit or mechanical ventilation or death, whichever occurred first.

COVID-19, coronavirus disease 2019; eGFR, estimated glomerular filtration rate.

^#^ vs GFR ≥ 90 ^&^ vs 60 ≤ GFR < 90.

Compared with the HEG, the MEG and LEG were associated with more frequent use of HFNC, NMW, IMV, and CRRT. The use of antibiotics, glucocorticoids, RAS inhibitors, and diuretics was also significantly higher in the MEG and LEG than in the HEG. The frequency of complications was higher in the MEG and LEG than in the HEG. In addition to secondary infection, other complications were more common among patients in the MEG and LEG than among those in the HEG; these included respiratory failure, ARDS, acute heart failure, acute cardiac injury, coagulation disorders, sepsis, shock, anemia, hypoproteinemia, electrolyte disturbances, and acidosis.

### Renal injury and in-hospital prognosis

On admission, 4.7% and 13.5% patients had elevated SCr and BUN, respectively; 30.5% patients had hematuria, and relatively fewer patients (19.1%) had proteinuria (Table 2). Only 5.3% and 1.9% patients had 2 + –3 + hematuria and proteinuria, respectively. Compared with patients in the HEG, patients in the MEG and LEG had elevated renal dysfunction indicators (SCr and BUN). In addition, hematuria and proteinuria at presentation were more common in the MEG and LEG than in the HEG (Table 2). During hospitalization, the peak SCr increased gradually with decreasing eGFR (Table 2). The incidence of AKI in the entire cohort was 6.7% according to KDIGO criteria (Table 3). Stage 1 AKI was present in 61.4% of the patients with AKI, while stages 2 and 3 were reached in 22.8% and 15.8% patients, respectively. The incidence of in-hospital AKI was higher in the MEG and LEG than in the HEG (Table 3).

**Figure 1:**
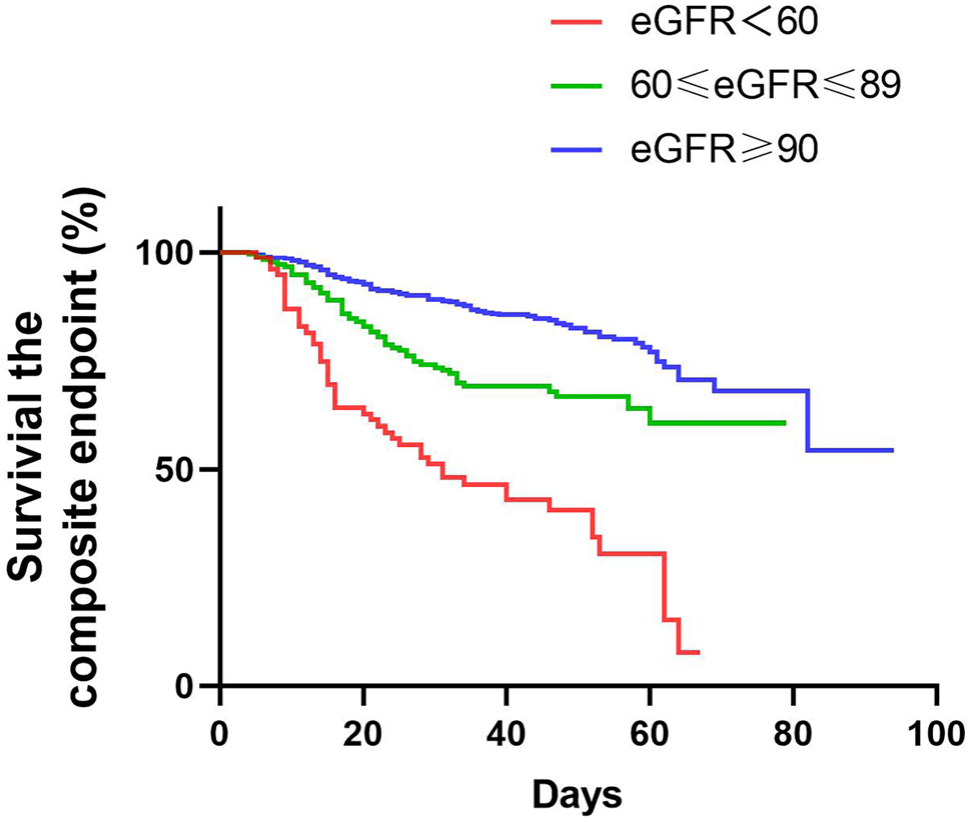
Kaplan–Meier survival curves for the composite endpoint among patients with COVID-19 stratified according to eGFR. COVID-19, coronavirus disease 2019; eGFR, estimated glomerular filtration rate.

**Figure 2:**
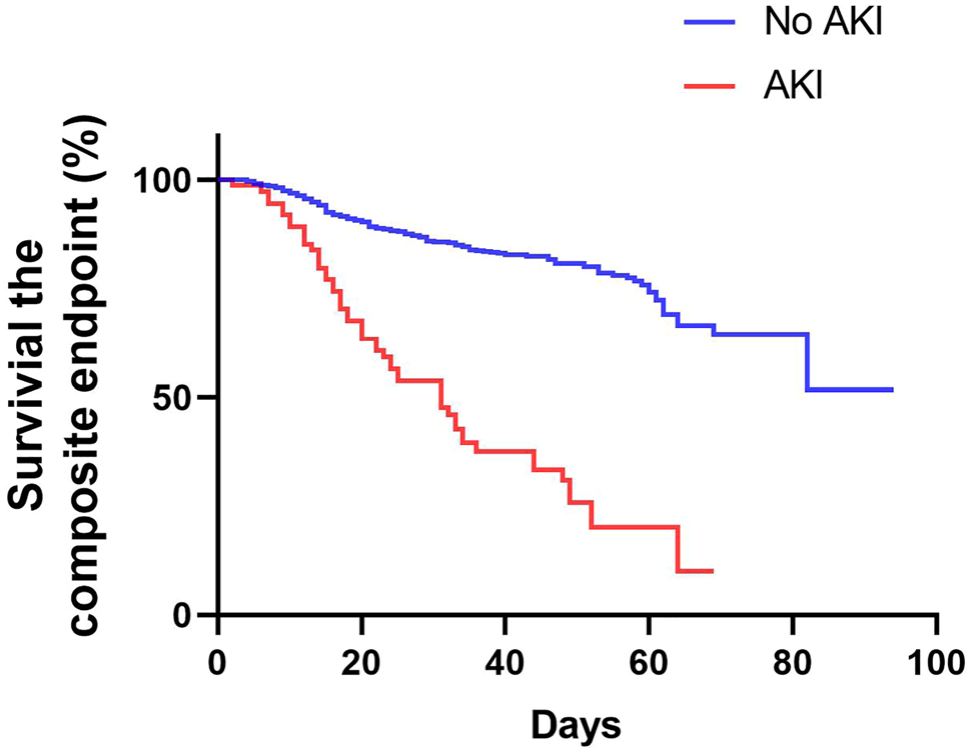
Kaplan–Meier survival curves for the composite endpoint among COVID-19 patients with and without AKI. COVID-19, coronavirus disease 2019; AKI, acute kidney injury.

A primary composite endpoint event occurred in 314 patients (18.6%), including 18.3% who were admitted to the ICU, 4.5% who underwent IMV, and 4.9% who died (Table 3). The median time from symptom onset to composite endpoint was 21 days (IQR, 13.0–33.5 days). Compared with those in the HEG, significantly more patients in the MEG and LEG reached the composite endpoint (15.4% vs. 19.6% vs. 54.5%; P = 0.000; Table 3). The rates of death (2.4% vs. 7.4% vs. 27.7%; P = 0.000), ICU admission (15.2% vs. 19.3% vs. 52.5%; P = 0.000), and IMV (2.9% vs. 6.3% vs. 18.8%; P = 0.000) also increased with decreasing eGFR. Compared to patients in the HEG, those in the MEG and LEG had shorter disease durations (from symptom onset to composite endpoint). Table 4 shows the comparison of characteristics and outcomes between patients with and without AKI. Patients with AKI yielded poorer in-hospital outcomes than those without, including the composite endpoint (46.9% vs. 9.4%; P = 0.000), the rates of death (32.1% vs. 2.4%; P = 0.000), ICU admission (44.3% vs. 9.3%; P = 0.000), and IMV (32.1% vs. 2.4%; P = 0.000). Kaplan–Meier survival analysis revealed that survival until the composite endpoint was significantly lower in the MEG and LEG than in the HEG (P = 0.000, Fig. 1). Patients with AKI had significantly escalated risks of reaching the composite endpoint compared with those without AKI (P = 0.000, Fig. 2).

**Table 4:** Clinical characteristics and outcomes of COVID-19 patients with or without AKI.

	AKI	no AKI	P value
N (%);	115 (6.7)	1593 (93.27)	-
Age (years);	69 (63,77)	62 (50,70)	0.000
Male sex, n/N (%);	60/115 (52.17)	758/1593 (47.58)	0.341
**Disease severity, n/N (%)**			
Non-severe	39/115 (33.91)	1080/1593 (67.80)	0.000
Severe	76/115 (66.09)	511/1593 (32.08)	0.000
**Comorbidities**			
Hypertension	69/115 (60.00)	577/1593 (36.22)	0.000
Diabetes	25/115 (21.74)	263/1593 (16.51)	0.148
Coronary artery heart disease	20/115 (17.39)	163/1593 (10.23)	0.017
Chronic kidney disease	10/115 (8.70)	33/1593 (2.07)	0.000
eGFR at admission	96.7 (78.0, 110.2)	100.8 (89.7, 112.4)	0.010
Any complication, n/N (%)	115/115 (100.00)	1235/1593 (77.53)	0.001
Day of hospitalization when AKI occurred (days)	8 (5, 14)	-	-
Death, n/N (%)	37/115 (32.17)	39/1593 (2.45)	0.000
ICU admission, n/N (%)	51/115 (44.35)	149/1593 (9.35)	0.000
Mechanical ventilation, n/N (%)	37/115 (32.17)	41/1593 (2.57)	0.000
Composite endpoint, n/N (%)	54/115 (46.96)	151/1593 (9.48)	0.000

**Figure 3:**
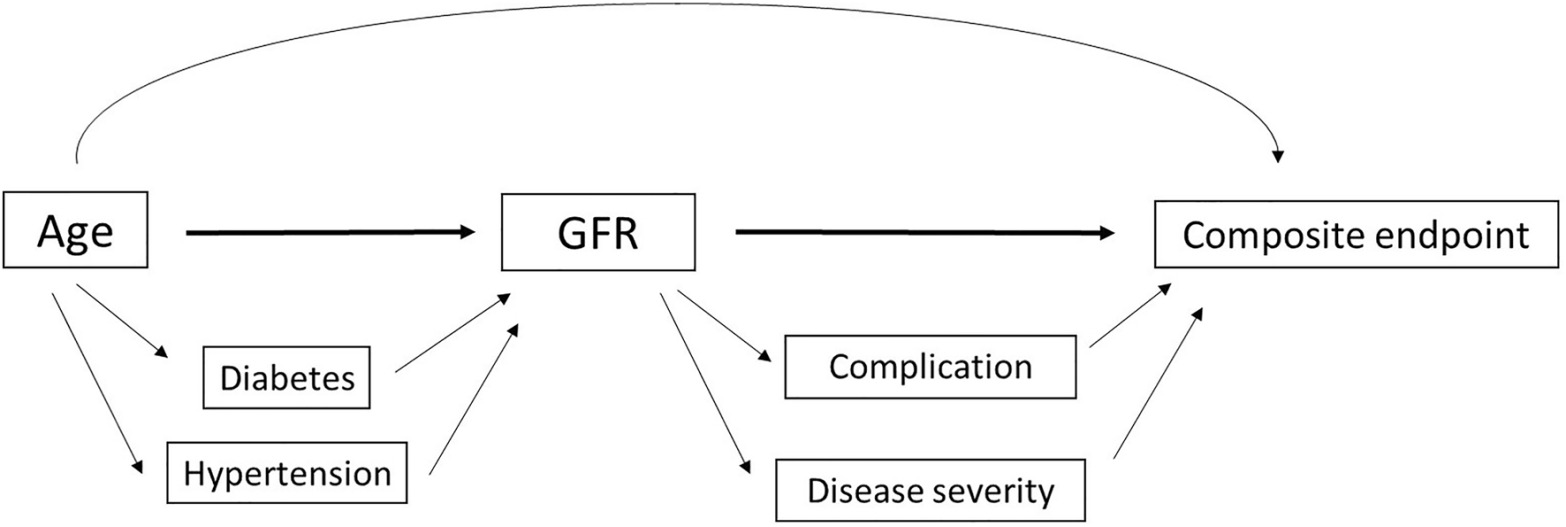
A directed acyclic graph (DAG) showing the association between risk factors and the poor prognosis of COVID-19.

Two models of multivariate analysis were calculated. The first (Model 1) was designed to address whether eGFR predicted outcomes independently of disease severity, preexisting disorders, and baseline clinical data. The second model (Model 2) was designed to address whether eGFR was an independent predictor of outcomes even when follow-up data during hospitalization were taken into account; this model included disease severity, preexisting disorders, and baseline and follow-up data. Model 1 showed a significantly higher risk of the composite endpoint (reference: HEG, ≥ 90 mL/min/1.73 m^2^; MEG, 60–89 mL/min/1.73 m^2^: HR = 1.538, 95% CI: 0.978–2.421, P = 0.063; and LEG, < 60 mL/min/1.73 m^2^: HR = 1.953, 95% CI: 1.174–3.250, P = 0.01; Table 5) and the mortality (reference: HEG; MEG: HR = 3.723, 95% CI: 1.565–8.854, P = 0.003; and LEG: HR = 7.382, 95% CI: 3.026–18.011, P < 0.001; Table 6) in the LEG than in the HEG and MEG. In addition, we found that eGFR was an independent predictor of outcomes in both 18–64 (non-elderly) and > 65 years old (elderly) groups. Patients aged 18 to 64 years had a 4.868-fold higher risk of the composite endpoint in the LEG group than those in the HEG group. The risk of the composite endpoints in LEG group was 2.379 times higher than that in HEG group in patients older than 65. The results indicated that renal impairment on admission was a greater predictor of poor prognosis in non-elderly patients than that in elderly patients (Table 5). Model 2 showed that relative to the patients in the HEG, the HR (95% CI) was 1.535 (0.976–2.413) (P = 0.064) in the MEG and 1.942 (1.168–3.230) (P = 0.011) in the LEG. In both models, AKI was an independent risk factor for the composite endpoint, with a high HR of 1.724 (95% CI: 1.070–2.778) (P = 0.025) in Model 1 and an HR of 1.650 (95% CI: 1.027–2.652) (P = 0.039) in Model 2 (Table 5). The results of the unadjusted analysis are presented in Tables 5 and 6. Overall, the findings of the unadjusted and adjusted analyses were not materially altered. A directed acyclic graph (DAG) showed the association between risk factors and the poor prognosis of COVID-19 based on the model variables in Cox regression (Fig. 3).

**Table 5:** Multivariate Cox regression analysis of associations of baseline eGFR and in-hospital AKI with the composite endpoint in patients with COVID-19.

Variable	Unadjusted HR (95% CI)	*p* value	Adjusted HR (95% CI)			
			Model 1^a^	*p* value	Model 2^b^	*p* value
**All patients**						
**eGFR, ml/min/1.73 m**^**2**^						
≥ 90	1.0 (Ref)		1.0 (Ref)		1.0 (Ref)	
60–89	2.066 (1.478–2.889)	0.000	1.538 (0.978–2.421)	0.063	1.535 (0.976–2.413)	0.064
< 60	5.595 (3.938–7.951)	0.000	1.953 (1.174–3.250)	0.010	1.942 (1.168–3.230)	0.011
**AKI**						
without AKI	1.0 (Ref)		1.0 (Ref)		1.0 (Ref)	
With AKI	5.263 (3.831–7.246)	0.000	1.724 (1.070–2.778)	0.025	1.650 (1.027–2.652)	0.039
**Patients aged 18–64**						
**eGFR, ml/min/1.73 m**^**2**^						
≥ 90	1.0 (Ref)		1.0 (Ref)		1.0 (Ref)	
60–89	1.491 (1.022–2.177)	0.038	1.231 (0.435–3.481)	0.485	1.502 (0.532–4.242)	0.442
< 60	3.490 (2.311–5.271)	0.000	4.868 (2.378–9.968)	0.000	4.327 (2.149–8.709)	0.000
**AKI**						
without AKI	1.0 (Ref)		1.0 (Ref)		1.0 (Ref)	
With AKI	2.525 (1.703–3.742)	0.000	1.954 (1.305–2.927)	0.001	1.727 (1.133–2.631)	0.011
**Patients aged ≥ 65**						
**eGFR, ml/min/1.73 m**^**2**^						
≥ 90	1.0 (Ref)		1.0 (Ref)		1.0 (Ref)	
60–89	1.486 (1.025–2.181)	0.038	1.164 (0.792–1.709)	0.440	1.080 (0.735–1.587)	0.696
< 60	3.471 (2.305–5.288)	0.000	2.379 (1.562–3.623)	0.000	2.133 (1.401–3.247)	0.000
**AKI**						
without AKI	1.0 (Ref)		1.0 (Ref)		1.0 (Ref)	
With AKI	1.824 (1.563–2.127)	0.000	1.542 (1.302–1.827)	0.000	1.470 (1.244–1.737)	0.000

^a^Model 1: adjusted for disease severity, hypertension, diabetes, coronary artery heart disease, cerebrovascular disease, leukocyte count, lymphocyte count, and IL-6.

^b^Model 2: adjusted for disease severity, hypertension, diabetes, coronary artery heart disease, cerebrovascular disease, leukocyte count, lymphocyte count, IL-6 and any complication (excluding AKI).

Composite endpoint comprised admission to the intensive care unit or invasive ventilation or death, whichever occurred first.

COVID-19, coronavirus disease 2019; eGFR, estimated glomerular filtration rate; AKI, acute kidney injury; HR, hazard ratio; CI, confidence interval; IL, interleukin.

**Table 6:** Multivariate Cox regression analysis of associations of baseline eGFR with the mortality in patients with COVID-19.

Variable	Unadjusted HR (95% CI)	*p* value	Adjusted HR (95% CI) ^a^	*p* value
**All patients**				
**eGFR, ml/min/1.73 m**^**2**^				
≥ 90	1.0 (Ref)		1.0 (Ref)	
60–89	3.399 (1.961–5.892)	0.000	3.723 (1.565–8.854)	0.003
< 60	11.155 (6.504–19.135)	0.000	7.382 (3.026–18.011)	0.000
**Patients aged 18–64**				
**eGFR, ml/min/1.73 m**^**2**^				
≥ 90	1.0 (Ref)		1.0 (Ref)	
60–89	0.992 (0.125–7.908)	0.000	1.459 (0.174–12.219)	0. 728
< 60	11.168 (3.377–36.933)	0.000	11.064 (3.055–40.065)	0.000
**Patients aged ≥ 65**				
**eGFR, ml/min/1.73 m**^**2**^				
≥ 90	1.0 (Ref)		1.0 (Ref)	
60–89	2.300 (1.240–4.266)	0.008	3.393 (1.184–21.960)	0.023
< 60	7.284 (3.897–13.617)	0.000	7.430 (2.514–9.725)	0.010

^a^adjusted for disease severity, hypertension, diabetes, coronary artery heart disease, cerebrovascular disease, leukocyte count, lymphocyte count, CRP, IL-6, and procalcitonin.

COVID-19, coronavirus disease 2019; eGFR, estimated glomerular filtration rate; HR, hazard ratio; CI, confidence interval.

## Discussion

In this multi-hospital observational study, we found that renal function was closely related to the prognosis of hospitalized COVID-19 patients. More than 25% of the patients had impaired renal function, with an eGFR lower than 90 mL/min/1.73 m^2^. Patients with abnormal eGFR (< 90 mL/min/1.73 m^2^) are more susceptible to severe COVID-19 infection. Although patients with impaired renal function received more intensive oxygen therapy, continuous blood purification, and glucocorticoid treatment, their prognosis was unsatisfactory, with a higher incidence of the composite endpoint (ICU admission, IMV, or death) and complications (AKI, respiratory failure, ARDS, acute cardiac injury, coagulation disorders, sepsis, shock, anemia, hypoproteinemia, electrolyte disturbances, and acidosis). Multivariate regression analysis showed that both eGFR < 60 mL/min/1.73 m^2^ on admission and AKI occurrence during hospitalization were independent risk factors for poor in-hospital prognosis.

We found that eGFR was an independent predictor of outcomes in both 18–64 (non-elderly) and > 65 years old (elderly) groups. The results showed that patients aged 18 to 64 years had a 4.868-fold higher risk of the composite endpoint in the LEG group than those in the HEG group. The risk of the composite endpoints in LEG group was 2.379 times higher than that in HEG group in patients older than 65 years. These results indicated that renal impairment on admission was a greater predictor of poor prognosis in non-elderly patients than that in elderly patients. So we drew the DAG diagram by taking age, eGFR and the compound endpoint as the main factors. Diabetes and hypertension can aggravate the progression of CKD and also lead to poor prognosis in patients with COVID-19^19,20^. So diabetes and hypertension are important confounding factors. Moreover, CKD patients had a higher risk of severe COVID-19^21^ and complications such as AKI^22^. In addition, our data showed that low eGFR was associated to the severe disease and the occurrence of complications. Therefore, eGFR may influence the outcome of patients with COVID-19 by regulating these two factors.

Serum SCr and BUN are commonly used to evaluate renal function, however, their levels are also affected by age, race, gender and body type. As the best overall index of kidney function by fully adjusting the effect of other factors on serum SCr, eGFR is the only criterion for staging CKD and determining long-term renal outcomes^23^. The normal eGFR is ≥ 90 mL /min/1.73m^2^; an eGFR of 60–89 mL/min/1.73 m^2^ indicates mild renal impairment; and an eGFR < 60 mL/min/1.73 m^2^ indicates moderate renal impairment; an eGFR < 15 mL /min/1.73m^2^ indicates renal failure and requires dialysis or renal transplantation. A study of a multicenter registry found that 30% of COVID-19 patients had kidney dysfunction upon admission (eGFR < 60 mL/min/1.73 m^2^), which was associated with higher in-hospital mortality^24^. It's important to note that eGFR at the admission may be reduced due to long duration of COVID-19 (25 days) in our study, but that this situation is similar for all the subjects. Our study showed that the incidence of complications, the composite endpoint (15.4% vs. 19.6% vs. 54.5%, P = 0.000), and mortality (2.4% vs. 7.4% vs. 27.7%, P = 0.000) increased gradually with decreasing eGFR. The risk of reaching the composite endpoint among patients in the MEG and LEG was 2.1 and 5.6 times, respectively, of the risk among those in the HEG (P = 0.000). This suggests that patients with renal function impairment (eGFR < 90 mL/min/1.73 m^2^) on admission had significantly worse in-hospital outcomes, although the significance was lost in the MEG (eGFR: 60–89 mL/min/1.73 m^2^) after adjustments for baseline and follow-up clinical data using the Cox regression model. Our results indicated that patients with an eGFR of 60–89 mL/min/1.73 m^2^ had already escalated risks of poor prognosis, emphasizing the need for early and continuous monitoring of renal function.

The prevalence of CKD in China is 10.8%^25^. However, in our study, only 2.6% of patients with COVID-19 had a history of CKD. We also found that 19.1% and 30.5% patients had proteinuria and hematuria, respectively. Thus, CKD may be underestimated in these patients on admission. Patients with kidney injury, especially those with GFR < 60 mL/min/1.73 m^2^, were less likely to develop fever, with an incidence of less than 50%. Despite a low incidence of clinical symptoms, patients with eGFR < 90 mL/min/1.73 m^2^ had a higher incidence of complications, the composite endpoint, and mortality. This may be due to low immunity (reduced lymphocyte count)^26,27^, coagulation disorders (decreased platelet count, prolonged prothrombin time, and increased d-dimer)^28,29^, poor nutrition (anemia and hypoproteinemia), and persistent inflammatory states (high procalcitonin, interleukin-6, and C-reactive protein)^30^ in patients with impaired renal function (eGFR < 90 mL/min/1.73 m^2^). In addition, anorexia on admission is a risk factor for poor prognosis (Supplement Table 2), which may be associated with subsequent malnutrition.

Acute kidney injury (AKI), as a common complication of COVID-19, is usually related to disease mortality^31^. A postmortem patient series found significant acute tubular injury in all patients who had died of COVID-19^32^. The mechanisms of kidney injury in SARS-CoV-2 infection include direct viral injury via the angiotensin-converting enzyme 2 receptor, which is highly expressed in the kidneys^33^, an imbalanced renin–angiotensin–aldosterone system^34,35^ and release of proinflammatory cytokines elicited by the viral infection and microvascular thrombosis^36^. We found that patients with eGFR < 60 mL/min/1.73 m^2^ were more likely to develop AKI during hospitalization, with an incidence of 17%. Furthermore, 10% of patients with eGFR < 60 mL/min/1.73 m^2^ received CRRT. A study in New York has reported a high incidence of AKI (37%–46%) among COVID-19 patients^32^ and 19% of patients with AKI required dialysis, and half of them died in the hospital. We found that AKI was an independent risk factor in patients with COVID-19 after adjustments. Therefore, regular monitoring of renal function and timely diagnosis of AKI are conducive to the treatment of COVID-19 patients.

Our study has some limitations. First, the incidence of CKD and AKI may be underestimated in some patients due to the lack of baseline medical records, late admission, and lack of renal function examination after admission. Second, a small proportion of patients were still in the hospital, and their outcomes were unknown at the time of the data cutoff, which might lead to the underestimation of the endpoint events. Third, due to different diagnostic paradigms in different hospitals, not all laboratory tests were performed in all patients, which led to some missing data. Last but not the least, there was no direct evidence of renal damage caused by the virus in the urine or kidney tissue.

In conclusion, impaired renal function on admission and the occurrence of AKI during hospitalization are independent predictors of poor prognosis among hospitalized COVID-19 patients. Therefore, early and continuous monitoring of renal function and early diagnosis of AKI are necessary interventions to predict and prevent the progression of COVID-19.

## Methods

### Study design and participants

This retrospective, multicenter study included 3 cohorts of 1851 adult in-patients (≥ 18 years) with confirmed COVID-19 pneumonia who were hospitalized in 3 hospitals that are designated care centers for patients with emerging infectious diseases in Wuhan (Tongji Taikang Hospital, Huo Shen Shan Hospital, and Renmin Hospital of Wuhan University) between February 3, 2020 and April 10, 2020. All patients were diagnosed with COVID-19 pneumonia according to WHO interim guidance^37^. Confirmed cases denoted patients with positive findings on high-throughput sequencing or real-time reverse-transcription polymerase chain reaction assays of nasal and pharyngeal swab specimens^38^. 28 Patients without renal function tests were excluded. Participants were followed up until discharge or in-hospital demise. This study was approved by the Research Ethics Commission of each participating site. Data collection was in accordance with the review board and therefore with all valid guidelines. Written informed consent was waived by the Ethics Commissions of the designated hospitals for emerging infectious disease.

### Data collection and definitions

Demographic characteristics, clinical data (symptoms, comorbidities, treatments, complications, and outcomes data), laboratory findings, and chest computed tomography (CT) findings were retrieved from electronic medical records by 4 investigators (F.X., Y.L., X.D.L, Y.Y., and Y.L). The eGFR was calculated using the Chronic Kidney Disease Epidemiology Collaboration Eq. ^39^. AKI was defined as an increase in SCr of ≥ 26.5 µmol/L within 48 h or a 50% increase in SCr from the baseline within 7 days, according to the Kidney Disease—Improving Global Outcomes (KDIGO) criteria^40^. Baseline SCr was defined as the SCr value on admission. The date of AKI onset was defined as the earliest day on which a SCr change meeting the KDIGO criteria was recorded. The stage of AKI was determined using the peak SCr level after AKI detection, with increases of 1.5–1.9, 2.0–2.9, and ≥ 3 times the baseline value being defined as AKI stages 1, 2, and 3, respectively. All cases were diagnosed and classified according to Interim Guidelines for COVID-19 of China (6th edition) provided by the National Health Commission of China. Clinical manifestations consist of four categories, mild, moderate, severe and critical. Mild cases were defifined as: (a) mild symptoms and (b) no abnormity on chest CT. Moderate cases were defifined as: (a) mild symptoms and (b) abnormalities on chest CT. Severe cases were defifined as either: (a) respiratory rate > 30 breaths/min, or (ii) oxygen saturation 93%, or (iii) PaO2/FiO2 ratio 300 mmHg. Critical cases were defined as including one criterion as follows: shock, respiratory failure requiring mechanical ventilation, organ failure requiring admission to ICU. Acute respiratory failure was defined as a decrease in oxygen saturation (< 92%) while breathing room air with severe respiratory distress or hypoxemia (partial oxygen pressure < 60 mm Hg) and/or requirement of invasive/noninvasive mechanical ventilation. Shock and acute respiratory distress syndrome (ARDS) were defined in accordance with WHO interim guidance^41^. Acute cardiac injury was diagnosed if serum levels of cardiac biomarkers (e.g., high-sensitivity cardiac troponin I) were above the 99_th_ percentile upper reference limit, or if new abnormalities were observed on electrocardiography and echocardiography^42^. Acute heart failure was defined as the clinical syndrome characterized by typical symptoms (e.g., breathlessness, ankle swelling, and fatigue) that may be accompanied by signs (e.g., elevated jugular venous pressure, pulmonary crackles, and peripheral edema) caused by a structural and/or functional cardiac abnormality^42^. Sepsis was defined according to Sepsis 3.0 or SIRS (Systemic Inflammatory Response Syndrome). Patients with septic shock can be identified with a clinical construct of sepsis with persisting hypotension requiring vasopressors to maintain MAP ≥ 65 mmHg and having a serum lactate level > 2 mmol/L despite adequate volume resuscitation^43^. It should be noted that the SIRS criteria may underestimate the real incidence of sepsis. Secondary infection was diagnosed when patients showed clinical symptoms or signs of pneumonia or bacteremia, and a positive culture of a new pathogen was obtained from lower respiratory tract specimens (qualified sputum, endotracheal aspirate, or bronchoalveolar lavage fluid) or blood samples after admission^5^. Anemia was defined as a hemoglobin level of < 120 g/L for male patients or < 110 g/L for female patients. Hypoproteinemia was defined as a blood albumin level of < 35 g/L.

### Statistical analysis

Categorical variables were expressed as counts and percentages, and continuous variables were expressed as medians with interquartile ranges (IQRs). Linear regression was used to perform collinearity analysis on variables related to outcomes. The Kruskal–Wallis test was applied to continuous variables, and the chi-square test and Fisher exact test were used for categorical variables, as appropriate. The composite endpoint was ICU admission, invasive ventilation, or death, whichever occurred first. Survival curves for the composite endpoint were derived using the Kaplan–Meier method, and differences between curves were analyzed using the log-rank test. Multivariate Cox regression models were used to test the associations of baseline eGFR and in-hospital AKI with the composite endpoint during hospitalization. The sensitivity of Model 1 and Model 2 was analyzed by the stepwise regression. The results are presented as hazard ratios (HRs) with 95% confidence intervals (95% CIs) and P values. All statistical analyses were performed using SPSS *v*22.0 (IBM, Armonk, NY, USA). For all analyses, P ˂ 0.05 (two-tailed) was considered significant.

## Supplementary Information

Supplementary Tables.
