# Supplementary material for: Clinical outcomes of hospitalized COVID-19 patients with renal injury: a multi-hospital observational study from Wuhan
Source: Sci Rep. 2021 Jul 26;11:15205. doi: 10.1038/s41598-021-94570-1 (PMC8313555; doi:10.1038/s41598-021-94570-1)
Supplement: Supplementary file 1 — Supplementary Tables. [file 41598_2021_94570_MOESM1_ESM.docx]

**Supplementary Table 1. Clinical Symptoms COVID-19 patients stratified by eGFR levels**

| **Variable** | **All patients**  **(N = 1851)** | **eGFR, ml/min/1.73 m^2^** | | | **P value^a^** |
| --- | --- | --- | --- | --- | --- |
|  |  | **≥90**  **(N = 1379)** | **60-89**  **(N=360)** | **＜60**  **(N=112)** |  |
| **N (%)** | 1851 | 1379 (74.5) | 360 (19.4) | 112 (6.1) |  |
| Fever | 1099/1851(59.37) | 838/1379(60.76) | 208/360（57.77） | 53/112（47.32）^#^ | 0.001 |
| Cough | 1044/1851（56.40） | 794/1379(57.57) | 196/360（54.44） | 54/112（48.21） | 0.112 |
| Sputum production | 309/1851（16.69） | 248/1379(17.98) | 48/360（13.33） | 13/112（11.60）^#^ | 0.036 |
| Fatigue | 766/1851（41.38） | 550/1379(39.88) | 166/360（46.11） | 50/112（44.64） | 0.080 |
| Shortness of breath | 491/1851（26.52） | 343/1379(24.87) | 108/360（30.00） | 40/112（35.71）^#^ | 0.011 |
| Chills | 253/1851（13.67） | 181/1379(13.12) | 53/360（13.72） | 19/112（16.96） | 0.424 |
| Hemoptysis | 100/1851（5.43） | 77/1379(5.58) | 18/360（5） | 5/112（4.46） | 0.821 |
| Headache/dizziness | 88/1851（4.75） | 65/1379（4.71） | 19/360（5.28） | 4/112（3.57） | 0.791 |
| Nasal congestion/[rhinorrhea](http://www.youdao.com/w/rhinorrhea/#keyfrom=E2Ctranslation) | 113/1851（6.10） | 88/1379（6.38） | 21/360（5.83） | 4/112（3.57） | 0.784 |
| Pharyngodynia | 236/1851（12.75） | 179/1379(12.98） | 45/360（12.5） | 12/112（10.71） | 0.778 |
| Myalgia/arthralgia | 192/1851（10.37） | 136/1379(9.86) | 42/360（11.66） | 14/112（12.5） | 0.455 |
| Chest pain | 43/1851（2.32） | 33/1379(2.39) | 8/360（2.22） | 2/112（1.78） | 1.000 |
| Chest distress/palpitations | 371/1851（20.04） | 257/1379 (18.64) | 90/360（25.00）^#^ | 24/112（21.43） | 0.025 |
| Anorexia | 238/1851（12.85） | 158/1379(11.45) | 66/360（18.33）^#^ | 14/112（12.5） | 0.003 |
| Diarrhea | 224/1851（12.11） | 165/1379(11.97) | 50/360（13.89） | 9/112（8.04） | 0.241 |
| Nausea/vomiting | 56/1851（3.02） | 40/1379(2.90) | 13/360（3.61） | 3/112（2.67） | 0.708 |
| Disturbance of consciousness | 8/1851（0.43） | 7/1379(0.51) | 0/360（0） | 1/112（0.89） | 0.230 |
| Asymptomatic | 38/1851（2.05） | 32/1379(2.32) | 4/360（1.11） | 2/112（1.79） | 0.039 |

Data are expressed as median (interquartile range) or no./total no. (%). ^a^P values were calculated using the Kruskal–Wallis test or the chi-square test.

COVID-19, coronavirus disease 2019; eGFR, estimated glomerular filtration rate; BMI, body mass index

^#^ vs GFR≥90 ^&^ vs 60≤GFR＜90

**Supplementary Table 2.** Multivariate Cox regression analysis of associations of clinical symptoms with the composite endpoint in patients with COVID-19

| Clinical symptoms | **HR (95% CI)** | **P-value** |
| --- | --- | --- |
| Fever | 1.23 (0.920-1.644) | 0.162 |
| Sputum production | 1.313 (0.861-2.002) | 0.205 |
| Shortness of breath | 1.193 (0.671-1.656) | 0.404 |
| Chest distress/palpitations | 2.104 (0.294-15.045) | 0.459 |
| Anorexia | 2.185 (1.443-3.309) | 0.000 |
| Asymptomatic | 1.608 (0.223-11.588) | 0.638 |
